# Advantages of CRISPR-Cas9 combined organoid model in the study of congenital nervous system malformations

**DOI:** 10.3389/fbioe.2022.932936

**Published:** 2022-09-02

**Authors:** Li Xiaoshuai, Wang Qiushi, Wang Rui

**Affiliations:** ^1^ Department of Blood Transfusion, Shengjing Hospital of China Medical University, Shenyang, China; ^2^ Department of Stem Cells and Regenerative Medicine, Key Laboratory of Cell Biology, National Health Commission of China and Key Laboratory of Medical Cell Biology, Ministry of Education of China, China Medical University, Shenyang, China

**Keywords:** organoid, CRISPR-Cas9, congenital nervous system malformation, central nervous system, 3D

## Abstract

In the past 10 years, gene-editing and organoid culture have completely changed the process of biology. Congenital nervous system malformations are difficult to study due to their polygenic pathogenicity, the complexity of cellular and neural regions of the brain, and the dysregulation of specific neurodevelopmental processes in humans. Therefore, the combined application of CRISPR-Cas9 in organoid models may provide a technical platform for studying organ development and congenital diseases. Here, we first summarize the occurrence of congenital neurological malformations and discuss the different modeling methods of congenital nervous system malformations. After that, it focuses on using organoid to model congenital nervous system malformations. Then we summarized the application of CRISPR-Cas9 in the organoid platform to study the pathogenesis and treatment strategies of congenital nervous system malformations and finally looked forward to the future.

## Introduction

In developed and developing countries, congenital disabilities are the leading cause of infant mortality and perinatal mortality (Baldacci et al., 2018). Congenital nervous system malformation (CNSM) is the third most common malformation after cardiovascular and renal system malformation. The deformity rate of CNSM is higher in people with high blood relatives: 2.3/1,000 in western countries, and 5.6/1,000 in Asian countries (BoAli et al., 2018). Most children require lifelong treatment and care, which has a severe impact not only on physiology but also on psychology. Therefore, it is essential to find out the pathogenic mechanism of CNSM during fetal neural development.

Innovative biological tools are an indispensable part of scientific progress. The success of any bioanalytical tool depends on its high precision, repeatability, cost performance, and practicality (Whiteley et al., 2022). Biotechnology can rarely have a significant impact in a short time, especially in genetics and development. The emergence of CRISPR-Cas9 and 3D organoid cultivation has wholly changed the present situation (Whiteley et al., 2022). The discovery of these two technologies can help us dig into the pathogenesis, diagnosis, and treatment of CNSM and other diseases.

## The occurrence of cnsm

During the whole pregnancy, the nervous system will experience rapid growth and development, and the evolution will continue from birth to the early stage of adulthood (Xu et al., 2021). The nervous system may be destroyed at any stage in the process of embryo development. Different times and locations of the damage will lead to varying types of CNSM (BoAli et al., 2018). The development of the brain and spinal cord begins with the neuroectoderm, and the subsequent development process is mainly divided into four stages. The first stage is the dorsal induction: the formation and closure of the neural tube, which occurs at the 3–5 weeks of pregnancy. If an abnormality occurs at this stage, it will lead to dorsal induction deformity, including anencephaly, encephalocele, cephaloceles, Arnold-Chiari malformation and spina bifida. The second stage is the ventral induction stage, which occurs at the 5–10 weeks of pregnancy and will differentiate into the cerebral hemisphere/thalamus, midbrain, cerebellum/brainstem, and face. If the development is abnormal, it will lead to ventral induced malformation, such as holoprosencephaly, Dandy-Walker malformation, cerebellar hypoplasia, Joubert syndrome, cerebellar hypoplasia/dysplasia, optic nerve dysplasia, pituitary abnormalities and facial abnormalities. The third stage is migration and histogenesis, which occurs at the 2–5 months of pregnancy. The main changes are the migration of neurons from the germinal matrix to the cortex and cortical tissue. If the migration fails, it will lead to abnormal gyration patterns, schizencephaly and corpus callosum hypoplasia. If the histogenesis fails, it will lead to microcephaly, macrocephaly, aqueductal stenosis, arachnoid cysts, neurocutaneous syndrome, congenital vascular malformation and congenital brain tumor. The fourth stage is myelination, occurring from the sixth month of gestation to the third year of pregnancy. The main changes are myelination from caudal to rostral, from posterior to anterior regions, and from central to peripheral locations. If the development fails, it will lead to diseases such as leukodystrophy, metabolic disorders and other metabolic disorders (BoAli et al., 2018).

## 3D organoid model of cnsm

The traditional two-dimensional (2D) co-culture method is a relatively simple method for reproducible multicellular models. It is an excellent platform that can be used to analyze cell-to-cell interactions. The 2D cell model can use primary cells or induced pluripotent stem cells (iPSC) to establish co-culture of multiple cell types (Figure 2). Although the 2D co-culture of cells is a relatively simple method to study the interaction between cells, it can’t deeply understand the complex pathophysiological process of diseases. In CNSM, 2D cell culture lacks the multiple cell types, long-distance interaction, the complexity and functionality of neural circuits *in vivo*. Therefore, we need a three-dimensional (3D) structure that can bring out the genetic and structural complexity of the nervous system to reveal the pathogenic process of CNSM disease (Ripke et al., 2014). The appearance of the organoid system as a 3D cell culture system helps to understand the process of tissue and organ development, and also helps to simulate various diseases of human beings, especially the nervous system diseases. The brain organoid is a 3D tissue model of autologous development derived from omnipotent or pluripotent stem cells, which can summarize the whole process of neurodevelopment, including neurogenesis, gliogenesis, synaptogenesis, cell migration, and cell differentiation (Lancaster et al., 2013; Lee et al., 2017; Vieira et al., 2021). Therefore, the brain and nervous system organoid provide an unprecedentedly complex *in vitro* model system for studying human-specific neural development and neural maturation (Lancaster et al., 2013; Vieira et al., 2021).

Brain organoids derived from iPSC of patients with neurodevelopmental disorders can reproduce the pathophysiological phenotype of diseases in dishes. In addition, It can show the characteristics of structural changes in embryonic neocortex cells caused by exposure to teratogens (such as viruses and drugs) during the perinatal period *in vitro* (Schnoll et al., 2021). Therefore, brain organoids can reflect not only hereditary factors but also the nervous system disease models caused by environmental factors. Next, we summarized the latest findings of the application of brain organoids based on iPSC in CNSM disease modeling, pathogenesis research, and related treatment strategies. At present, most studies on CNSM organoids focus on microcephaly and macrocephaly.

### Microcephaly

Microcephaly, a congenital autosomal recessive hereditary disease, is a CNSM characterized by small brain volume and capacity, particularly affecting the size of the cerebral cortex (Faheem et al., 2015; Kristofova et al., 2022). There are 12 genes related to microcephaly, most of which encode centrosome proteins, which play an essential role in mitosis (Faheem et al., 2015). Among these genes, CDK5RAP2 regulates centriole replication, and the deletion of CDK5RAP2 has been shown to affect the proliferation of neural precursor cells, mainly by delaying chromosome segregation and chromosome instability (Gonzalez-Martinez et al., 2021,^2022^). However, mice with CDK5RAP2 mutation did not show severe brain volume reduction in human patients (Gonzalez-Martinez et al., 2021). Lancaster et al. constructed brain organoids mimicking microcephaly used iPSCs from microcephaly patients with CDK5RAP2 heterozygous nonsense mutations (Lancaster et al., 2013). The results showed that compared with the control group, the brain organoids of patients showed smaller neuroepithelial areas and changed the spindle direction of radial glial cells. Notably, the brain organoids of patients are smaller in size. Further research shows that the small size results from damaged proliferation and expansion of neural progenitor cell bank and premature differentiation of neurons (Florio and Huttner, 2014; Gonzalez-Martinez et al., 2021). Before neurogenesis, the neural progenitor cells of mice do not continue to proliferate and expand as humans do, which explains that CDK5RAP2 deficient mice do not show the same microcephaly as humans (Florio and Huttner, 2014; Gonzalez-Martinez et al., 2021). Another example of using brain organoids to model microcephaly is Aicardi Goutieres syndrome (AGS). AGS is characterized by severe neuron loss, leading to lifelong disability (Adang et al., 2020; Aditi et al., 2021). The pathogenic genes of this disease are diverse. Seven gene mutations, including TREX1, RNASEH2B, and RNASEH2C have been identified to cause AGS (Rice et al., 2013). The lack of a reliable animal model of AGS hinders the research of disease physiology and treatment (Onizawa et al., 2021). Therefore, when Thomas et al. used iPSCs derived from AGS patients to study brain organoids, they found many nerve cell death and nerve cell inflammation, which is similar to clinical neurodegeneration and eventually leads to microcephaly (Thomas et al., 2017). Recent studies also have shown that overexpression of PTEN results in reduced neural precursor proliferation, premature neuronal differentiation, and markedly reduced size in human brain organoids, ultimately leading to microcephaly (Dhaliwal et al., 2021).

### Macrocephaly

Macrocephaly is a congenital developmental disorder. There are 27 syndromes associated with macrocephaly. It characterized by a secondary increase in the size and/or number of neurons and glia. It causes overgrowth and increased size of the brain. The brain eventually stops growing prematurely, resulting in abnormal brain development (Juric-Sekhar and Hevner, 2019; Zhang et al., 2020). These changes are most apparent in the prefrontal cortex manifested by increased cortex thickness and surface area (Juric-Sekhar and Hevner, 2019; Zhang et al., 2020). Studies on brain organoids derived from iPSC of patients with megacephaly found that neural progenitor cell (NPC) division increased significantly, and the differentiation was blocked. These changes eventually led to a significant expansion in organoid volume, which was consistent with the clinical manifestations of patients (Zhang et al., 2020). Dang et al. also confirmed that human cerebral cortex organoids with giant brain malformation showed delayed differentiation of neurons (Dang et al., 2021). It is worth noting that the results between NPC differentiated from patient-derived iPSC and brain organoids have been independently verified (Zhang et al., 2020; Dang et al., 2021). There is continuity and correlation, which indicates that this correlation is stable in brain organoids under the same genetic conditions (Zhang et al., 2020; Dang et al., 2021). In a recent study, macrocephaly and microcephaly phenotypes were observed in cortical organoids generated from iPSCs of patients with Copy Number Variation (CNV) in the 16p11.2 region (Urresti et al., 2021). CNV in 16p11.2 region affects not only the size of the organoids but also the maturation, the proliferation of neurons, and the number of synapses (Urresti et al., 2021). These findings prove that 3D organoid culture can provide a platform for researching pathogenic mechanisms and drug screening of various CNSM.

### Others

At present, the research on other organoid models of CNSM is limited. According to reports, RAD9B is a related gene associated with spina bifida, and its deletion is associated with DNA damage response (DDR). In the study of neural organoids formed by human embryonic stem cells (ESC) with RAD9B deletion, it is found that cell proliferation, differentiation, and cell adhesion are damaged compared with the control group, which is consistent with the phenotype of spina bifida. These results represent that DDR can be used as target for human neural tube defects (NTDs) (Cao et al., 2020). It is reported that congenital forebrain developmental malformation (such as total forebrain or cortical developmental malformation) is caused by an abnormal Sonic Hedgehog (Shh) signaling pathway, which is mainly manifested in functional primary cilia. Microtubule-based organelles are present in nearly every cell. Donovan et al. discussed the relationship between abnormal cilia function and forebrain diseases (such as total forebrain or cortical developmental malformation) (Donovan and Basson, 2017). It is emphasized that the developmental malformation of the forebrain should not only be studied through the animal model but also through organoid. Therefore, all these studies show that the brain organoid is an innovative tool to study CNSM, which is very similar to the environment of the human brain and can be used as a 3D model to study the development of nerves and the brain. However, if we want a deeper understanding of the pathogenic mechanism of different types of CNSM, we need to edit it at the genome level.

## Application Crispr-Cas9 to study the pathogenesis of cnsm on 3D organoid platform

**FIGURE 1:**
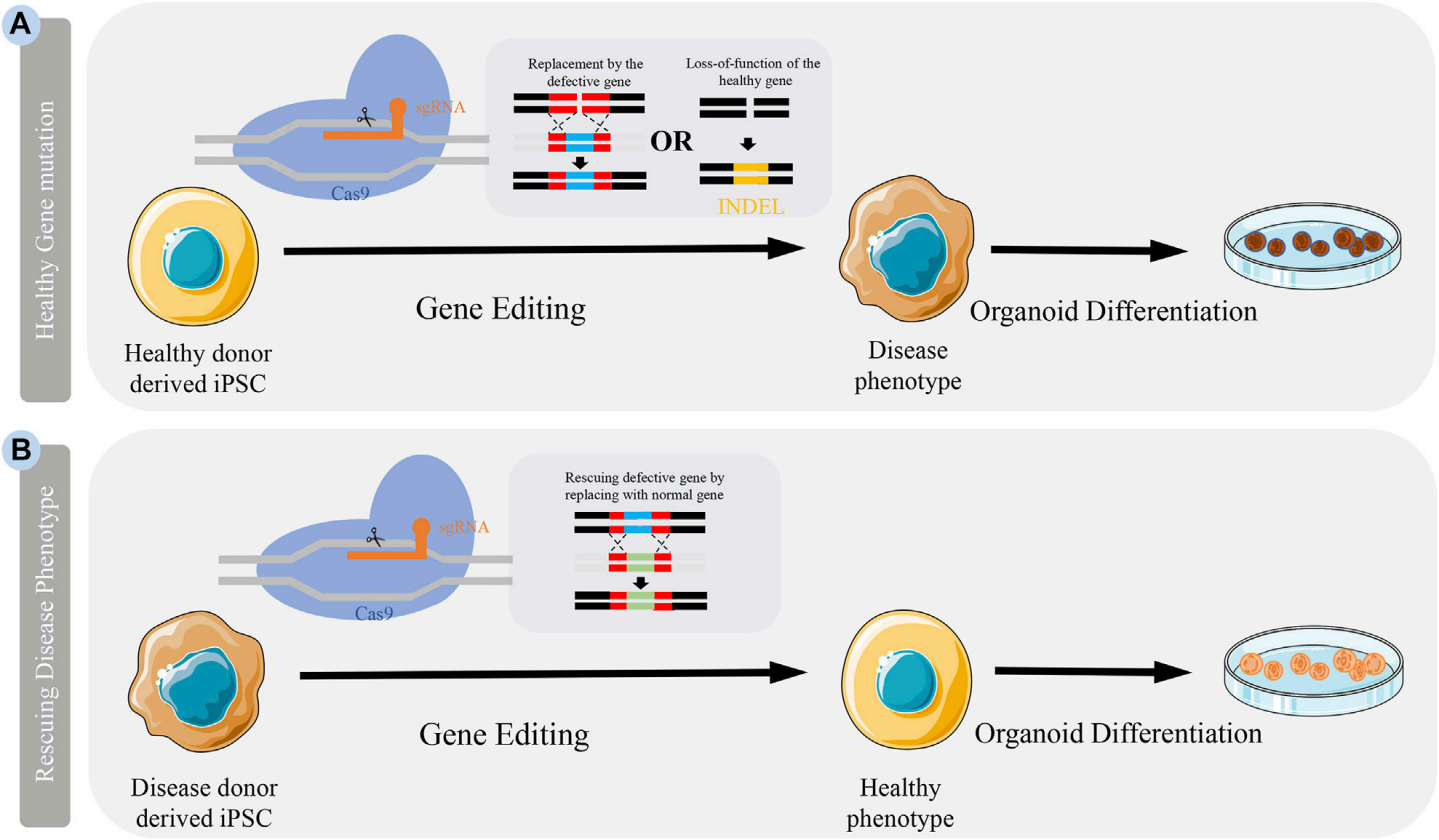
Application CRISPR-Cas9 to generate disease model. Models can be generated by either knocking out a healthy gene or rescuing a defective gene in cells. **(A)** Somatic cells from healthy people are reprogrammed into iPSCs. Specific disease-causing mutations can be introduced to genes in these cells by either knocking in defective genes or introducing INDEL through genome editing. **(B)** Somatic cells from patients can be isolated and reprogrammed into iPSCs. The mutation in these patient iPSCs can be corrected by the delivery of CRISPR-Cas9 introducing a nomal gene.

The simplest method to explore gene function is to re-edit the coding sequence of the gene so that it becomes non-functional. There are three genome editing technologies, namely zinc finger nucleases (ZFN), transcription activator-like effector nucleases (TALEN), and CRISPR-Cas (Gupta et al., 2019). In contrast to ZFN and TALEN, CRISPR gives it a great advantage due to its ease of operation. Among the CRISPR-Cas system, CRISPR-Cas9 is widely used because of its simple design, specificity and reduction of off-target editing (Karimian et al., 2019). It can be designed to recognize a specific sequence of DNA by recognizing a single-guide RNA (sgRNA) encoding an exon. While Cas9 cleaves DNA through two nuclease domains (HNH and RuvC), resulting in double-strand breaks (DSB) at the target site. The host cell responds to DSB with two different mechanisms: 1) Nonhomologous end joining (NHEJ) and 2) homology-directed repair (HDR) which lead, respectively to insertion or deletion (INDEL) and frameshift mutation in target DNA and HDR that offers a donor DNA as template for homologous recombination (Gasiunas et al., 2012; Karimian et al., 2019) (Figure 1). Currently, based on CRISPR, a more accurate single base editor-basic editor (BE) has been discovered. BE includes cytosinebase editor (CBE) and adenosinebase editor (ABE). CBE can change C G to T A, ABE can change A T to G C. BE retains the advantage of CRISPR-Cas9 system, and mainly transforms Cas9 scissors (Komor et al., 2016a; Gaudelli et al., 2017). Unlike Cas9, BE is mainly used to treat diseases caused by single-base mutations such as premature aging and sickle-cell anemia, and is also emerging as a promising approach to treat genetic diseases (Komor et al., 2016a; Anzalone et al., 2020). As a powerful genetic tool, CRISPR-Cas9 is widely used in life science research, which recently awarded the Nobel Prize in Chemistry (Cohen, 2020). At present, CRISPR-Cas9 has been applied to some iPSC-based CNSM models (Table 1). To better understand CNSM, it is necessary to study whether the physiological and pathological changes are produced by editing the target gene sequence of normal people or repairing the pathogenic genes of patients with diseases (Figure 1).

**TABLE 1:** Application of brain organoid models and/or CRISPER**-**Cas9 in CNSM.

Cell type	Gene	Disease model	Patient-derived (Y/N)	CRISPER-Cas9(Y/N)	References
iPSC	CDK5RAP2	Microcephaly	Y	N	Lancaster et al. (2013)
iPSC	TREX1	Aicardi Goutieres syndrome	Y	N	Thomas et al. (2017)
iPSC	PTEN	Microcephaly	Y	N	Dhaliwal et al. (2021)
iPSC	STRADA	Macrocephaly	Y	N	Dang et al. (2021)
iPSC	16p11.2 region	Macrocephaly and microcephaly	Y	N	Urresti et al. (2021)
ESC	RAD9B	Spina bifida	N	N	Cao et al. (2020)
iPSC	RAB39b	Macrocephaly	Y	Y	Zhang et al. (2020)
iPSC	FMR1	Macrocephaly	N	Y	Brighi et al. (2021)
ESC	OCLN	Microcephaly and cortical malformation	N	Y	Bendriem et al. (2019a)
iPSC	CPAP-E1235V	Microcephaly	Y	Y	An et al. (2022)
iPSC	SURF1	Leigh syndrome	Y	Y	Inak et al. (2021)
iPSC	HEXB	Sandhoff disease	Y	Y	Allende et al. (2018)

### Microcephaly

A Central dysfunction is related to microcephaly. To better understand the etiology of microcephaly, iPSC carrying the mutant protein gene-encoding centrosome CPAP-E1235V was generated by CRISPR-Cas9. The phenotypic characteristics of brain organoids derived from wild-type and mutant iPSCs were examined. The results demonstrate that the combination of iPSC-derived brain organoids and CRISPR-Cas9 can exhibit microcephaly pathological features and explain the possible mechanism of microcephaly (An et al., 2022). It is reported that the mutation of Occludin (OCLN) can lead to microcephaly and cortical malformation in humans. Studies by Bendriem et al. have demonstrated that CRISPR-Cas9 knockout the gene OCLN of mouse and human ESC and found that the brain organoids showed early neuronal differentiation disorder, slow self-renewal of progenitor cells, and increased apoptosis, and the human neural progenitor cells were more seriously affected (Bendriem et al., 2019a). Therefore, OCLN is needed to regulate the organization and dynamics of the centrosomes in early cerebral cortex formation, which reveals the new role of this tight junction protein in early brain development (Bendriem et al., 2019a).

### Macrocephaly

The mutation of the small GTPase gene RAB39b is related to congenital macrocephaly and intellectual disability (Zhang et al., 2020). Using CRISPR-Cas9 to knockout the gene RAB39b in iPSCs, Zhang et al. observed hyperproliferation and increased size of NPCs in human brain organoids (Zhang et al., 2020). Therefore, the authors conclude that cerebral cortical neurogenesis is altered due to RAB39b mutations, leading to macrocephaly and autism-like behavior (Zhang et al., 2020). Fragile syndrome X (FXS) is a congenital neurodevelopmental disorder, characterized by intellectual disability and sensory deficits, and megacephaly (Hagerman et al., 2017; Brighi et al., 2021). It is caused by epigenetic silencing of the gene FMR1 and the loss of its protein product FMRP. To study the role of FMRP proteins in brain development, Brighi et al. used CRISPR-Cas9 to knock out the FMR1 gene to generate brain organoids (Brighi et al., 2021). Compared with the control, the brain organoids derived from FMR1 knockout showed an increase in the number of glial cells, abnormal differentiation of nerve cells, and larger organoid size (Brighi et al., 2021).

### Others

Leigh syndrome (LS), also known as subacute necrotizing encephalomyelitis, is a hereditary progressive neurodegenerative disease (Inak et al., 2021). Models of LS have been generated by using iPSC from LS patients with the mutation gene SURF1 to cultivate brain organoids (Inak et al., 2021). Combined with CRISPR-Cas9, to correct the mutation gene SURF1 of iPSCs, the restoration to normal morphology can be observed through organoid culture (Inak et al., 2021). This provides insights into the pathogenesis and potential intervention strategies (Inak et al., 2021).

Sandhoff disease is a congenital disorder of neurometabolic (Allende et al., 2018). The content of GM2 ganglioside in the brain tissue of children is 100–200 times higher than usual, which leads to neurodegeneration and even death. Allende et al. produced iPSC from fibroblasts of infants with Sandhoff disease, then using CRISPR-Cas9, the mutated gene HEXB in iPSC was corrected by template repair and developed brain organoids to mimic the early neural development (Allende et al., 2018). Results showed that compared with HEXB-corrected organoids, brain organoids from Sandhoff disease accumulated GM2 gangliosides, which eventually led to brain development damage, indicating that GM2 gangliosides deposition affected the function of nerve cells in the early stage of nerve development (Allende et al., 2018). Therefore, CRISPR-Cas9 can find the pathogenic genes causing CNSM based on brain organoids, or even compensate for the pathogenic gene and reverse the disease.

## Conclusion

In this review, we first introduced the occurrence of CNSM, discussed the 3D modeling methods of CNSM, and introduced the research progress of 3D brain organoids in CNSM. Then we summarized the application of CRISPR-Cas9 in the organoid to study the pathogenesis, diagnosis and treatment strategies of CNSM, paying particular attention to the ability of brain and nerve development in different models.

**FIGURE 2:**
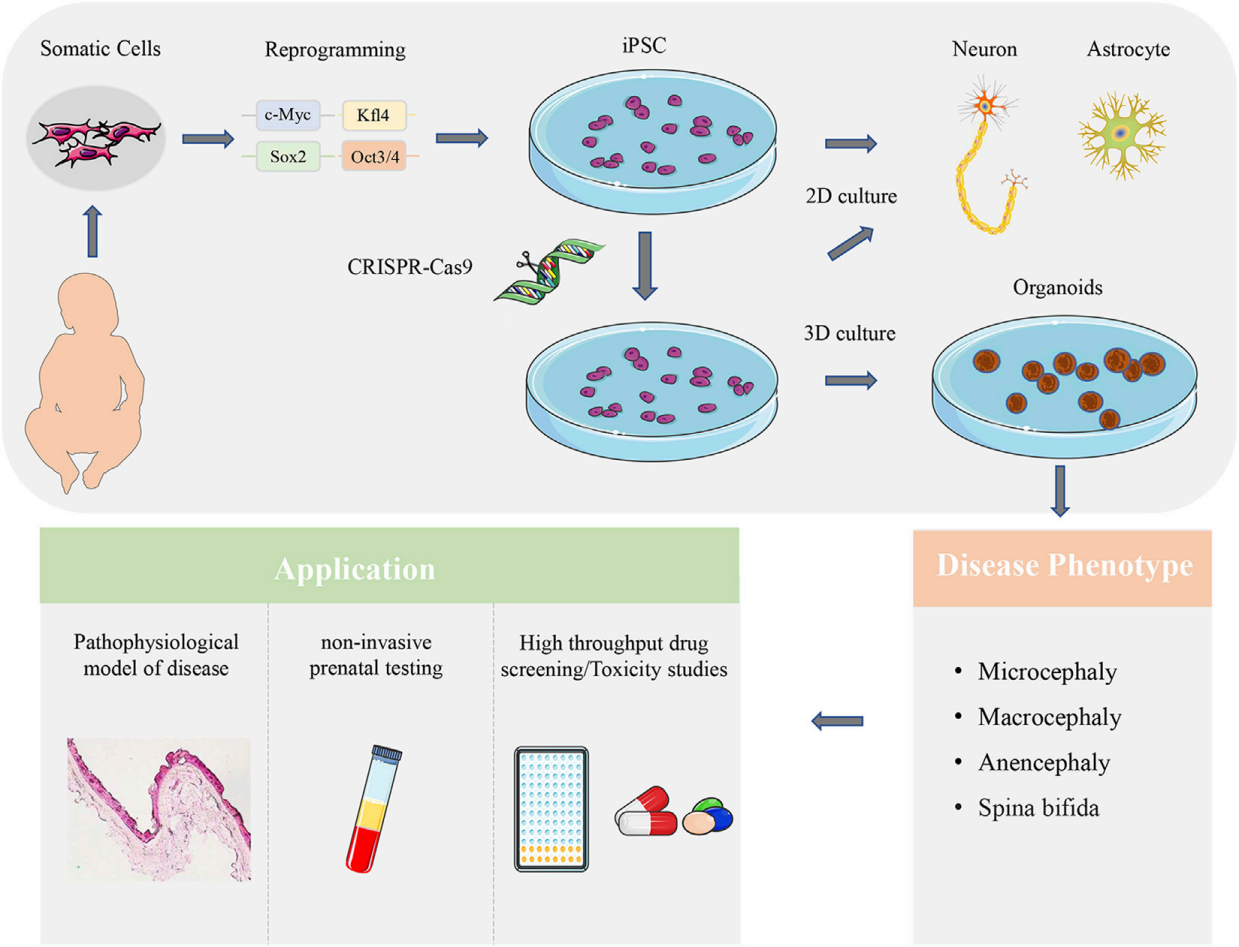
Application of brain organoid in CNSM. Patient-derived somatic cells reprogrammed into iPSCs can be differentiated into neurons and glial cells. The 2D co-culture models can analyze the interaction of different brain cell types. CRISPR-Cas9 can also be used to edit iPSCs, and observe the related characteristics of brain organoids, so as to determine the related mechanism of disease occurrence. Brain organoid can be used in different CNSM models to analyze the pathology of diseases, evaluate the efficacy of newly developed compounds in drug screening and toxicity research, and make non-invasive prenatal testing clinically.

Brain organoid can be used in different CNSM models to analyze the pathology of diseases, evaluate the efficacy of newly developed compounds in drug screening and toxicity research, and make non-invasive prenatal testing clinically (Figure 2). However, there is still much room for further optimization. Although the transcription regulators, embedding materials, and culture media in the culture process are straightforward, there are still differences among different batches of brain organoids, including cellular heterogeneity (Bhaduri et al., 2020). Automated culture methods can improve repeatability and homogeneity while maintaining the complexity of brain organoid structure (Renner et al., 2020). To truly understand the causes of different types of CNSM, a gene-editing technique is needed to establish phenotypes consistent with human models from the gene level to the transcriptome and cell physiology level *in vitro* research.

Compared with the traditional gene-editing technology, CRISPR-Cas9 has a higher gene-editing efficiency, lower off-target effect, and no DNA integration, so it is an ideal gene-editing technology (Yang and Yang, 2021). In addition, Some CNSM is caused by multiple genes, so it is necessary to repair multiple genes in order to completely reverse the disease. But so far, CRISPR-Cas9 is mainly used to repair single gene in CNSM. For example, in Sandhoff disease mentioned above, CRISPR-Cas9 corrected one of the pathogenic genes, finally relieved GM2 ganglioside, and even finally reversed the brain development damage (Allende et al., 2018). Furthermore, CRISPR-Cas9 has the potential to edit not only a single gene but also multiple genes simultaneously (Gupta et al., 2019). At present, in the study of the treatment of hepatitis B virus (HBV), there have been an example of CRISPR-Cas9 knockout multiple genes at the same time (Kato et al., 2021). However, it has not been reported in the study of CNSM. We hope that in the near future, the application of CRISPR-Cas9 can edit multiple genes simultaneously to reverse the diseases.

At present, CRISPR-Cas9 system has two important delivery systems to target cells, including viral and non-viral delivery systems (physical delivery systems). In the virus delivery system, two recombinant viruses (adeno-associated viruses (AAVs) and lentivirus) are more popular. Compared with viral delivery system, non-viral delivery system has its own advantages, including low cytotoxicity, transient expression and rapid action. In non-viral delivery system, electroporation is defined as a suitable tool for delivery of CRISPR-Cas9 system. In this review, electroporation is applied in almost all cases. However, CRISPR-Cas9 still has some limitations. One of the limitations is when CRISPR-Cas9 is used to insert foreign DNA into the target genetic locus, the efficiency of HDR is very low (Hussmann et al., 2021). Increase HDR by including inhibition of NHEJ related processes to make accurate DNA insertion (Hussmann et al., 2021). Researchers have also applicated the Cas9 nickel enzyme to increase HDR and improve the efficiency of Cas9 gene insertion (Ran et al., 2013). In addition, the off-target effect is also common, resulting in the editing of the genetic code at the non-target locus (Moon et al., 2019). Designing the CRISPR RNA carefully is essential to reduce the off-target effect. And the larger CRISPR construction size has a negative impact on the transfection rate (Mali et al., 2013). Overcoming these challenges can give full play to the potential of CRISPR-Cas9, and combining it with brain organoid can better explore the genetic complexity of CNSM.
